# Evaluation of family and staff experiences with virtual rounding and bedside presence in a tertiary neonatal intensive care unit during the COVID-19 pandemic

**DOI:** 10.1177/1357633X221081294

**Published:** 2022-03-29

**Authors:** Ashley Blagdon, Dani Smith, Tara Bramfield, Amuchou Soraisham, Ayman Abou Mehrem

**Affiliations:** 1 Department of Pediatrics, 70401 Cumming School of Medicine, University of Calgary, Calgary, AB, Canada; 2 26634 Foothills Medical Centre, Alberta Health Services, Calgary, AB, Canada; 3 Alberta Children's Hospital Research Institute, Calgary, AB, Canada

**Keywords:** Telemedicine, neonate, virtual communications, COVID-19 pandemic, family integrated care

## Abstract

**Introduction:**

The COVID-19 pandemic-related visitation restrictions started in March 2020 in Alberta, Canada. In the Neonatal Intensive Care Unit, we implemented a Virtual Communications initiative to allow parents to continue to be present with their infants, attend daily rounds, and communicate with the medical team. The purpose of this survey study was to describe our approach and evaluate the experience for families and staff.

**Methods:**

The study surveys consisted of 13–18 questions directed toward understanding staff and family experience with the process and emotional impact using Likert scale and open-ended questions. The study team reviewed results and implemented changes in real time. Analysis was mixed quantitative and qualitative design, with descriptive data organized into themes.

**Results:**

Twenty-six surveys were completed by 16 staff (62%) and 10 parents (38%). About 50% to 100% of respondents agreed or strongly agreed with statements addressing the quality and value of the virtual sessions. Staff identified challenges with slow devices and need for awareness and education. Both staff and parents expressed gratitude for the initiative and an overall positive experience.

**Discussion:**

Offering Virtual Rounds and Bedside Presence in the Neonatal Intensive Care Unit is a well-received and feasible alternative to in-person presence that allows parents to stay involved and connected to their infants. Families have a better understanding of their babies’ clinical status and plans with an overall positive experience.

## Introduction

Family centred care (FCC) is a framework approach that focuses on enabling parents to participate in the planning and delivery of healthcare to their child.^1^ Parental presence and involvement in their child's care greatly contributes to improved patient and family outcomes.^2–5^ Family Integrated Care (FICare) is an extension of the principles of FCC that has been implemented by Neonatal Intensive Care Units (NICUs) across the province of Alberta in Canada.^2,6–8^ In addition to improving infant outcomes such as reduced length of stay, the aim of the Alberta FICare model is to promote parent–infant connections, build parent confidence, and improve parent mental health through relational communication, education and support.^8^ Barriers such as parental illness, transportation issues, or work commitments can impede the delivery of Alberta FICare and alternative arrangements are not often readily available.

The public health and institutional restrictions associated with the onset of the SARS-CoV-2 (COVID-19) pandemic have resulted in an increasing number of parents and caregivers who are unable to enter the NICU. They cannot see or interact with their infants or participate in discussions and care plans with the healthcare team. Families and healthcare providers are already describing the significant impact of the COVID-19 pandemic restrictions on parental mental health and well-being, and disruption to parent integration in care, education, and transition to home.^9^

To ensure that families remain active members of the care team, the NICU at the Foothills Medical Centre (FMC) in Calgary, Alberta launched a Virtual Communications initiative. Parents can see and speak with their infants and healthcare providers or attend and participate in the daily multidisciplinary rounds using video- and audio-enabled devices. Similar work in virtual health prior to the pandemic had demonstrated parental satisfaction and interest.^10,11^ These efforts highlighted some crucial considerations when implementing this type of service, most notably technical challenges, staff buy-in, and the importance of support at organizational, operational, and human resources levels.^10,11^

An increasing number of healthcare institutions worldwide have embarked upon and described their experiences with adaptation of virtual technologies during the COVID-19 pandemic.^12,13^ Comprehensive description and evaluation of such Virtual Communications initiatives in the NICU may be helpful for other institutions wishing to launch or improve upon similar initiatives.

The purpose of the present study was to describe our institutional approach and evaluate the overall experience for staff and families with Virtual Communications in a Level III NICU in Calgary. Our goal was to understand the user perspective and inform quality improvement efforts to attain an effective and sustainable alternative to face-to-face communication when in-person presence is limited.

## Methods

### Setting and virtual communications process development

The FMC is a tertiary perinatal centre that provides services to all premature deliveries less than 32 weeks, high-risk obstetrical deliveries, and critically ill neonates in the southern part of the province of Alberta. The NICU is comprised of 39 Level III beds with average of 1100 admissions annually, including a mix of inborn and outborn infants. Out of these, approximately 120 infants are born less than 29 weeks gestation.

**Figure 1.**
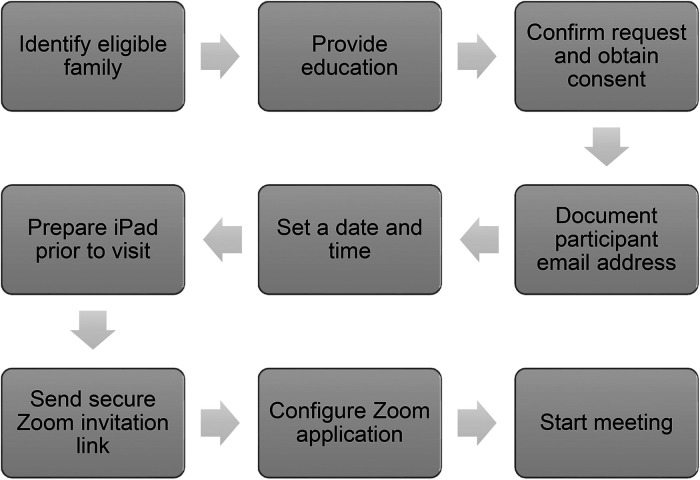
Virtual communications process flow diagram.

Alberta Health Services (AHS) first announced visitation restrictions to hospitals in relation to COVID-19 on 18 March 2020 – permitting one ‘well’ visitor at a time and no children. At this time, there was no alternative for parents and caregivers of infants admitted to the NICU. While the FMC NICU team leadership, collaborating with the Parent Advisory Council Team (PACT), hurried to find a virtual solution, AHS announced acquisition of an enterprise license for Zoom for all organizational members on 25 March 2020. Development for the Virtual Communications process began and representatives from the Legal and Privacy Department were engaged to ensure adherence to organizational standards such as the privacy and confidentiality principles from the Health Information Act and the Freedom of Information and Protection of Privacy Act as well as AHS virtual health policies (document available internally only).^14,15^ The first approved and configured tablet (an iPad) was provided by AHS for staff to use in the NICU on 30 April 2020 and the process was soft launched. Families wishing to participate required their own personal device. After further modifications to the process and troubleshooting which included obtaining software updates and new protective cases that enabled better audio and video quality, the NICU received one additional requested device from AHS and was able to implement the process to include the entire clinical care team with an official launch date of 28 May 2020 – over 2 months after the first visiting restrictions were announced (see Figure 1). Educational materials for staff and families such as informational handouts and a short video with frequently asked questions and how to troubleshoot technical difficulties were developed and distributed by the project team which included the study authors.

### Study design

Mixed methods quantitative and qualitative questionnaires were developed for this study using Qualtrics^XM^ Software through the University of Calgary. Two separate versions were created, one for staff and one for families, about their recent experience with Virtual Communications in the NICU in the format of multiple choice, 5-point Likert scales, and free-text responses (see Appendices 1 and 2). General topics of interest included: participant demographics, ease of use and challenges with devices and technology, usefulness of educational handouts, burden to workflow and daily life, overall satisfaction and feedback, likelihood of using again or recommending to other parents. Staff and parents were asked about their level of confidence using Virtual Communications, if they received education, information, or support, and to rate their level of agreement with statements about process and impact on a 5-point Likert scale. They were also asked to provide comments to elaborate on their responses. Additional topics of interest for parents included: understanding medical issues and care plans, ability to participate in planning and decision making, and ability to interact and bond with infant. Finally, parents were asked to rate the degree to which they experienced a list of positive and negative emotions on a 4-point scale including ‘not at all’, ‘very little, ‘somewhat’, and ‘very much’, as directly related to their Virtual Communications session.

### Ethics

The study protocol including process for informed consent and questionnaire was screened using the A pRoject Ethics Community Consensus Initiative Tool and it was determined to be ‘Low Risk’.^16^ A formal letter of exemption from the University of Calgary Conjoint Health Research Ethics Board review was obtained. It was also presented to and approved by the FMC NICU Parent Advisory Council Team (PACT) as well as the Calgary Zone Neonatal Quality Improvement Committee and the questionnaire was validated by pilot testing with members of PACT. Participants provided informed consent by reviewing the purpose, expectations, and potential risks and benefits of the study and proceeding to submit in completion. No personal identifying data was collected for research purposes and participants could withdraw from the study at any time. Personal information including email addresses was collected and managed in accordance with AHS guidelines and standards. Study investigator's contact information was provided. There was no incentive to participate.

### Participants

The target populations for this survey study included: (1) parents and caregivers of infants admitted to the NICU, and (2) NICU staff, including physicians, nurses, nurse practitioners, and allied health professionals. Inclusion criteria included usage of any form of Virtual Communications in the NICU (i.e. Virtual Multidisciplinary Team Rounds, Bedside Presence, or other Meeting) and ability to read and understand English.

### Data collection

All families and staff who used Virtual Communications in the NICU and met the inclusion criteria were invited to complete the survey. Dissemination occurred via email of a secure link along with a brief description of the project or Quick Response (QR) code. Individuals were invited to complete the survey after any participation respective to that session and there was no restriction on the number of times an individual could respond. There was no expiration for the link or QR code but once the survey was started participants had 1 week to submit.

### Data analysis

Responses were collected for 6 months from December 2020 to June 2021, with interim analysis at 2 and 4 months. Quantitative results for Likert scale questions determined agreement as either ‘strongly agree’ or ‘agree’ responses. Qualitative free-text answers were transferred verbatim to Microsoft Excel and inductively analysed by the primary investigators as described in the Association for Medical Education in Europe Guide No. 131.^18^ Descriptive data was organized by topic into major and minor themes with key direct quotations extracted. Emerging themes were discussed and reviewed by the study team during interim analysis to identify areas in need of immediate improvement.

## Results

### Participant information

A total of 26 complete responses were received from 16 staff (62%) and 10 parents (38%). Key demographics are shown in Table 1. Most staff and parents evaluated Virtual Bedside Presence with their infant (*n*  =  20; 77%), as opposed to Virtual Multidisciplinary Team Rounds (*n*  =  5; 19%), and one parent did both. Parents were asked to select any reason for their participation in Virtual Communications. Of note, four parents chose ‘illness in self or family other than infant’, three selected ‘other children to care for’, and zero selected ‘preferred to in-person visit’.

**Table 1.** Survey participant demographics.

Demographic	Participants (*n* = 26)
*Staff survey (n* *=* *16)*	* *
Clinical role	
* Nurse*	15 (94%)
* Pharmacist*	1 (6%)
Level of clinical experience	
* 5 years or less*	8 (50%)
* 6 years or more*	8 (50%)
Times participated in NICU VC	
* One*	6 (38%)
* Two*	2 (12%)
* Three or more*	8 (50%)
Type of VC	
* Bedside presence*	13 (81%)
* Rounds*	3 (19%)
*Parent survey (n* *=* *10)*	* *
Age of participant	
* *24 years old or less	1 (10%)
* *25 to 34 years old	5 (50%)
* *35 years old or more	4 (40%)
Relationship to infant	
* *Mother	9 (90%)
* *Father	1 (10%)
Infant GA at birth	
* *25 weeks or less	4 (40%)
* *26 to 29 weeks	3 (30%)
* *30 weeks or more	3 (30%)
Infant age at time of VC	
* *First week of life	3 (30%)
* *Second week of life	2 (20%)
* *During or after third week of life	5 (50%)
Times participated in NICU VC	
* *One	4 (40%)
* *Two	2 (20%)
* *Three or more	4 (40%)
Type of VC	
* *Bedside presence	7 (70%)
* *Rounds	2 (20%)
* *Other meeting	1 (10%)
Reason for participating^a^	
* *Live far from hospital	6 (60%)
* *Illness (self or other)	4 (40%)
* *Other children to care for	3 (30%)
* *Pandemic-related restrictions	2 (20%)
* *Work	2 (20%)
* *Financial issues	2 (20%)
* *Transportation issues	1 (10%)
* *Preferred to in-person	0 (0%)

^a^ Total percentages not equal to 100% as participants selected all that apply.

### Survey responses

**Figure 2.**
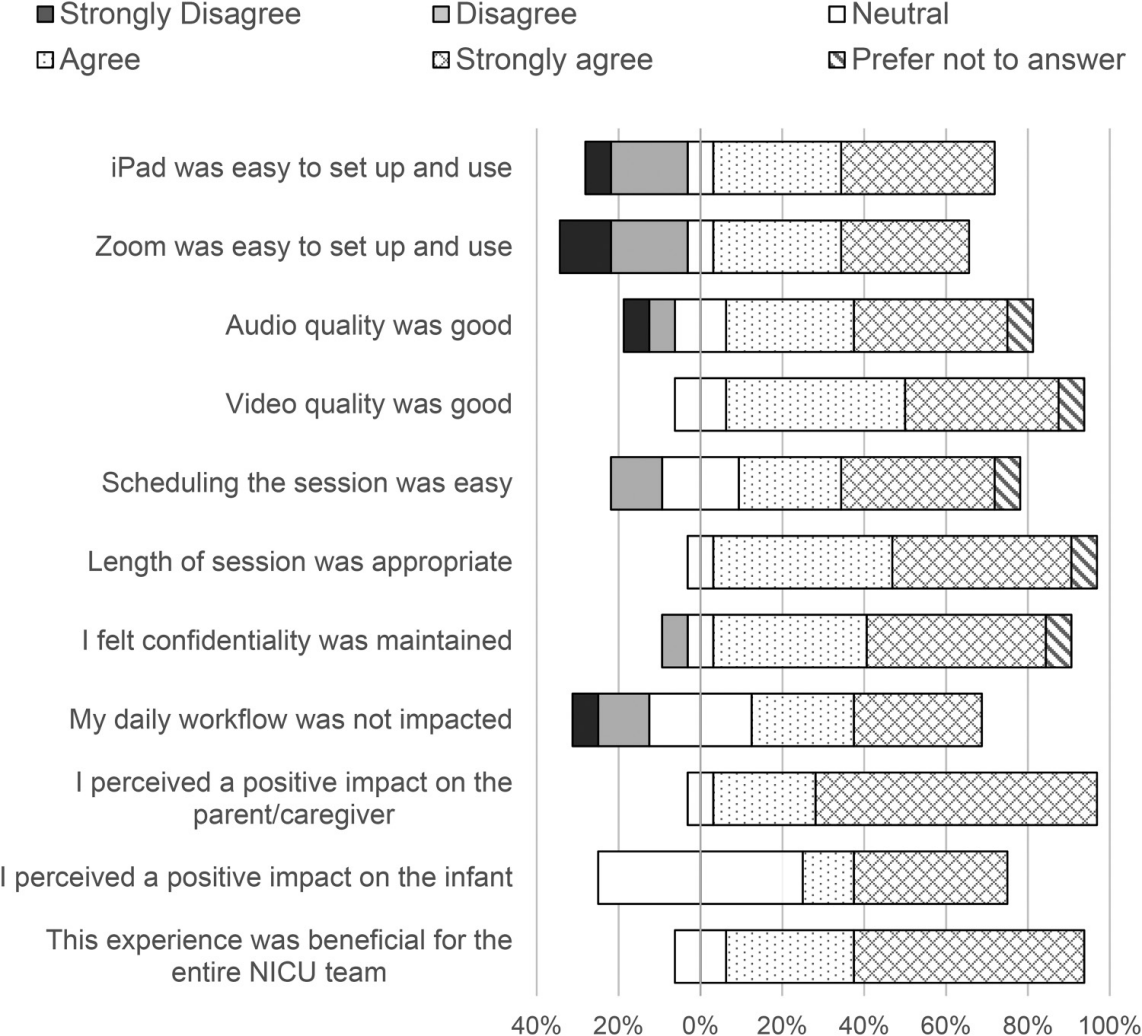
Staff responses to Likert scale questions. The percentages of respondents who agree with the statement are shown to the right of the zero percentage line; the percentages who disagree are shown to the left; the percentages who neither agree nor disagree are split down the middle.^17^

**Figure 3.**
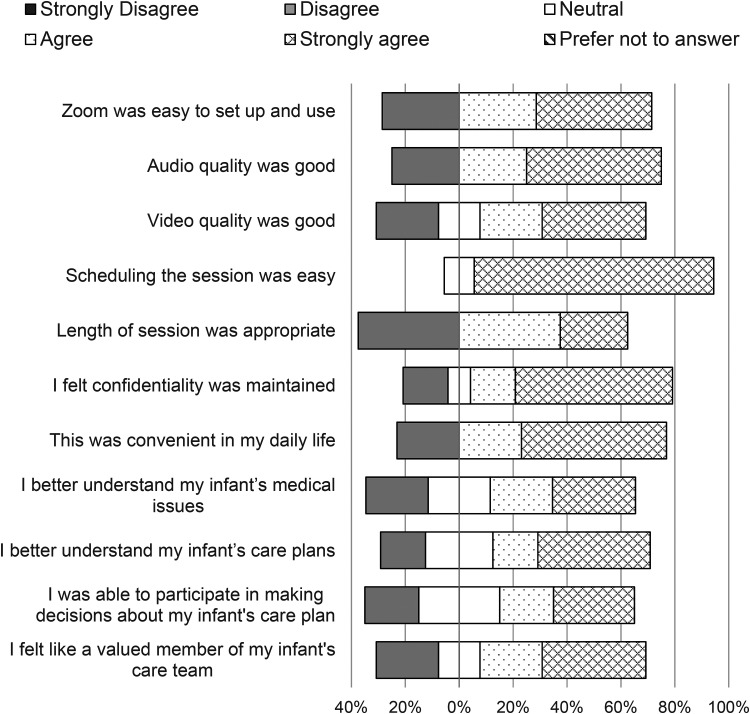
Parent responses to Likert scale questions. The percentages of respondents who agree with the statement are shown to the right of the zero percentage line; the percentages who disagree are shown to the left; the percentages who neither agree nor disagree are split down the middle.^17^

Staff responded to 11 statements on the quality and value of the virtual sessions. Agreement level indicated by responding with ‘agree’ or ‘strongly agree’ for each statement ranged between 50% and 94% of respondents, with an average agreement rate of 73% (Figure 2, Appendix 3). Parents responded to 12 similar statements with an agreement rate of 83%, ranging from 50% to 100% of respondents agreeing per statement (Figure 3, Appendix 3). The statement with which staff had the highest rate of agreement was, ‘*I perceived a positive impact on the caregiver’*, and the lowest rate of agreement was with, ‘*I perceived a positive impact on the infant’.* There were three statements with which parents agreed the most, including, ‘*Zoom was easy to set up and use’, ‘Length of session was appropriate’,* and ‘*This was convenient in my daily life’.* The statement parents agreed with the least was, ‘*During [Virtual Communications] I was able to participate in making decisions about my infant's care plans’.* When parents did not agree, they most often responded ‘neutral’ and ‘disagree’ was only selected four times. Most of the staff were ‘extremely’ (38%) or ‘fairly’ (44%) confident with using Virtual Communications and 69% of all respondents had received education or information prior to use (see Appendix 4). Eighty-eight per cent of staff and 90% of parents replied that they had an ‘excellent’ or ‘good’ experience and 100% of parents said it met their expectations. Hundred per cent of all survey respondents selected ‘yes’, they would recommend Virtual Communications to other families.

Thematic analysis of free-text comments lead to the identification of *Process* and *Impact* as the two major themes in both survey groups as presented in Tables 2 and 3. Sub-themes included *Identifying barriers, Suggestions for improvement, General comments about use,* and *Positive* and *Negative impacts* (staff did not make any comments about negative impact). The main barriers staff identified were issues with set up and connection, and suggestions included increasing awareness and education for all users, and technical improvement strategies like configuring wireless internet hotspots within the NICU. Parents commented less on process barriers, possibly because while staff were faced with connection issues with the hospital internet server, parents likely used home internet or cellular network technology. Parents similarly recognized the need for further staff awareness and education. The other suggestion was to consider a 24 h live feed in the NICU of each baby.

**Table 2.** Themes from staff survey free-text comments.

Themes	Participant comments		
	Sub-themes		
Process	*Identifying barriers*	*Suggestions*	*General comments*
	‘Setting up the zoom call was a bit complicated and confusing. It took some time to set it up’.	‘…as long as we can work out the technical side’.	‘I read the instructions manual’.
	‘My workflow was interrupted due to difficulties obtaining ZOOM and setting up’.	‘Usually, bedside nurses are confident in using it if we show them once’.	‘From my experience, it was easy to organize and use’.
	‘…more than one instance where I have sent the link as per the outlined process and the parent did not receive it’.	‘Bedside nurses should remind the parents that if parents can't visit in person every day, virtual visits or virtual rounds are always an option for them’.	‘It is a great tool and option to have’.
	‘It took forever to connect to the internet and one out of three iPads were not properly configured also the family couldn’t hear me or the other person very well’.	‘Maybe if we had a NICU email for FaceTime for apple users? or a NICU email that could be used to send photos to parents who are unable to visit’.	‘As a clinician, I usually help the bedside nurse set [up] the iPad and start the conversation. Then, I leave it to the bedside if I feel that the bedside nurse is confident in using it’.
	‘Couldn't get iPad to connect. Reset several times. Could do first 2 steps of setting up zoom mtg, but 3rd step stalled each time’.	‘If it could be more reliable, it would be even better! Can this inconsistency in links being sent be looked into and fixed?’	‘I replied yes to recommending virtual connections as I think its a great idea’.
	‘It would be helpful if we had easier access for contact with parents who are unable to visit, the process of setting up a zoom meeting can be long’.	‘…get a secure hot spot for the iPad so technical issues won’t be there’.	
	‘… never were able to connect’.		
	‘Internet connection was poor’.		
Impact	*Positive*	* *	* *
	‘I feel that when parents can attend rounds they have a better understanding of the plan and can provide input’.		
	‘…it was a very positive experience for the parents and the healthcare team…we had a baby on isolation for a long period of time…parents used virtual visits at least 2 times a day. I felt really good about the fact that we had this technology’.		
	‘There was no other way to communicate with that parent while maintaining confidentiality. This helped a lot - better for staff and parents’.		
	‘Parents really appreciated being able to see their baby when they could not come in’.		
	‘What a great tool for our population. I wish we could have started something like this sooner’.		

**Table 3.** Themes from parent survey free-text comments.

Themes	Participant comments		
	*Sub-themes*	*** ***	*** ***
Process	*Identifying barriers*	*Suggestions*	*Reason for use*
	‘The video was good but the sound was very shotty so it was very hard to hear what they said cause it cut in and out’.	‘I would suggest every quarantine room have an [iPad]. And have it set up so the nurses can quickly invite the parents online… have a designated IT person…’	‘…would use this as a way to see baby and let baby hear our voices not really a way to communicate with the nurses…’.
		‘Make it easier for the nurses to set up and use’.	‘We just use [Virtual Communications] to see her’.
		‘…a 24 hour feed of your baby’s bed that doesn't include sound so that it remains private for others in the NICU and [doctors] and nurses, would be an exceptional service..’.	‘I thought it was a great option considering I was too sick to travel to Calgary and also have 6 other children in my care’.
		‘[Nurses] just need to learn more about it’.	‘It was great to have VC as a way to see [and] talk to our baby when not able to be there with them’.
Impact	*Positive*	*Negative*	* *
	‘…this is very helpful to us at this time…we appreciate every ounce of effort that you guys put into helping us see our daughter daily and the amount of information during rounds so we [know] everything that is happening with her!’	‘While it was nice to see him unfortunately it's not incredibly intimate when so many others are around. It's a great option but it doesn't compare to being there in person’.	
	‘The virtual visits made me feel like I was a part of my baby's daily care since I was unable to physically visit her’.	‘It was hard to see him but not be able to touch him or comfort him’.	
	‘Since my whole family has covid things have been overwhelming, the VC has given me that sense of calm that my baby is doing ok based on my visual assessment of her. If she looks happy to me that makes me happy’.	‘At the beginning of all this it was very hard emotionally…’	
	‘Was excited to see my baby every visit’.		
	‘It was great to have VC…The nurse was great [at] getting close [to] the baby [so] we could talk to her’.		

Most comments from both groups of respondents about perceived impact of using Virtual Communications were descriptions of extremely positive experiences for the healthcare team and parents. While few parents provided insight into the emotional difficulty of not being physically present, feedback was mostly encouraging about the benefits of and gratitude for the opportunity to see and speak with their babies’ and being able to remain a part of the daily care plans.

**Figure 4.**
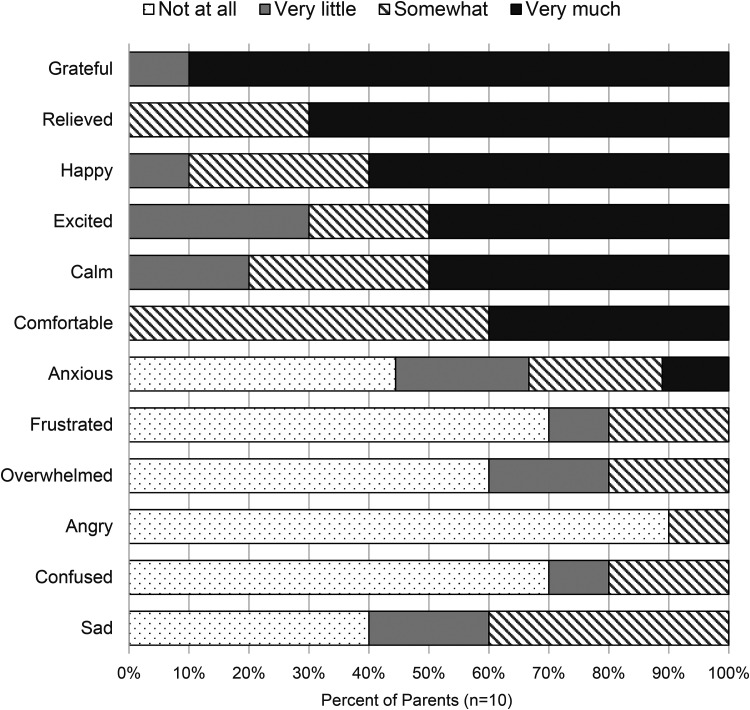
Parent emotional experiences during virtual communications in the NICU.

Lastly, when parents were asked to rate different emotions as directly related to their Virtual Communications session, they selected *grateful*, *relieved*, and *happy* as the most frequently experienced and *angry*, *overwhelmed*, and *frustrated* as the least frequently experienced (Figure 4).

## Discussion

The negative impacts of the COVID-19 pandemic are recognized globally and have affected even the most vulnerable infants in the NICU and their families.^9^ Our study findings were consistent with work done by others in this area – that Virtual Communications is a well-received and feasible alternative for parents and caregivers who are unable to physically be present such as during times of visiting restrictions.^9–13^ Additionally, our study comprehensively explored and helped leadership understand parent and staff experiences with Virtual Communications and assisted with improving the process in real time.

Detection of issues with the tablets very early in the process based on staff responses allowed leadership to quickly obtain higher quality devices to overcome this barrier to implementation. We were also informed about challenges with slow connection and time required to set up that was affirmed by the low number of respondents who took part in Virtual Rounds compared to Bedside Presence. This was partially addressed by upgrading devices and increasing support to frontline staff as indicated by improved feedback in subsequent surveys, but it has not been overcome completely and there is further work to be done. We also identified that although staff expressed feeling generally confident with process, many had not received education, information, or support. Thus, our team developed a brief video for staff to refer to as needed. We established that there was a positive overall experience for staff despite technical issues as almost all respondents ‘strongly agreed’ that there was a positive impact on the caregiver and that it was beneficial for the entire team. This evidence will be extremely useful to the sustainability of Virtual Communications in the NICU. We hope that it will encourage local staff and leadership that the time and resources required to support this service are valuable.

Parents were generally satisfied with the Virtual Communications process and there were no significant issues identified. As in staff surveys, we were alerted to the internal challenges with devices and connection. Most parents received education, information, or support, which confirmed that frontline staff were doing an excellent job. We were very encouraged that the overwhelming majority of parents reported either an ‘excellent’ or ‘good’ overall experience with Virtual Communications, and every respondent indicated that they would recommend the service to other families. We determined an overall successful endeavour in that most parents were better able to understand their infant's medical issues and care plans and felt like a valued member of the care team with participating in decisions and feeling a bond with their infants’. However, many parents indicated they were ‘neutral’ about these efforts, and a few expressed that they were not able to participate in decision making for their infant's care plans. As we did not identify a similar finding in the literature, this may require further exploration in future studies. In our context, we believe this result may have been influenced by individual motivation for use as some parents indicated they only wanted to see and interact with their baby, to know that they are doing well. Determining that each parent has unique expectations and needs during Virtual Communications has allowed us to emphasize the importance of individualizing the experience and establishing goals prior to each session. We found it very reassuring that no parent responded that they preferred Virtual Communications as we did not wish to discourage in-person presence. Similar to staff, more parents had participated in Virtual Bedside Presence, and further work is required to understand and address associated barriers to Virtual Rounds.

Finally, most parents expressed experiencing positive emotions related to using Virtual Communications. We were encouraged that a large proportion felt *grateful*, *relieved*, *happy, excited, calm, and comfortable*. Very few parents expressed feeling negative emotions such as *sad, confused, angry, overwhelmed,* or *frustrated*. Based on free-text comments, we determined that the parents who did feel these emotions ‘somewhat’ may have felt so in relation to being separated from their baby or seeing them in a NICU environment. Overall, this is a reassuring outcome of the survey indicating that our primary aims are being achieved.

In our experience, we identified three principal lessons that might be helpful to other organizations. First, organization-wide timely and effective communication strategies and information-sharing are crucial. In addition to minimizing time and resources wasted by duplicate efforts, this may have avoided extraneous worry and frustration by reassuring all departments within the hospital that tools and resources would soon be available. Second, organizational preparedness and adaptability in time of crisis is paramount. The recognition of a shared vision and acceptance of new technologies to support family presence during times of visitation restriction were appreciated as a priority. Organizational leadership support was a critical rate-limiting step in propelling this innovation forward. Previous requests to use Zoom had been denied due to organizational risks that were subsequently mitigated through proper process development ensuring privacy and safety of all participants. Third, access to resources and funding is time sensitive. Lack of readily available resources and funds to support process development and device acquisition was another barrier to implementation. Requests were submitted then triaged resulting in slow procurement of devices for the NICU; delaying process launch by nearly 2 months. Large organizational investments in technology including telehealth platforms and devices as well as teams dedicated to support the process would significantly improve time to implementation.

### Limitations

One of limitations of this study is the small sample size. Secondly, while the survey tool was validated, responses could have been influenced by external factors such as individual user interpretation and technical issues with completing the survey online. Furthermore, although our results indicate overall positive user experiences, there may be bias in participant selection as online survey respondents may be more technologically-savvy, English-speaking, and potentially of higher socio-economic and educational status. Finally, other institutions may find little use in the description of our approach if it does not fit within the policies and practices of their organization.

## Conclusion

The development and implementation of Virtual Communications in a Level III NICU was an onerous but invaluable process. Through the dissemination and analysis of surveys to staff and parent participants, we have demonstrated that offering this service is deeply appreciated to fulfil a variety of needs including allowing parents to see and interact with their infants when in-person presence is not possible, and to remain an active member of the medical care team. This information has been used to expand implementation of Virtual Communications across Level II NICUs in Calgary. We hope that this comprehensive description and evaluation will inspire other institutions wishing to embark or improve upon similar initiatives, and that it will be useful at a local organizational level to keep Virtual Communications a sustainable process that we can continue to offer families long into the future.

## Appendix 1. Staff Survey Questions

1. How many times have you participated in Virtual Communications in the NICU (including the most recent time)?
  - 1
  - 2
  - 3
  - 4 or more
2. In which type of Virtual Communication in the NICU did you most recently participate?
  - Bedside Virtual Visit
  - Virtual Rounds
  - Other Virtual Meeting (specify if possible)
3. What is your clinical role in the NICU?
  - Registered Nurse
  - Nurse Clinician
  - Respiratory Therapist
  - Fellow/Resident/Nurse Practitioner
  - Neonatologist
  - Other/Allied Health (please specify)
4. Please indicate your level of experience within your clinical area:
  - 0 to 2 years
  - 3 to 5 years
  - 6 to 9 years
  - 10 to 19 years
  - 20 years or more
5. How confident were you using Virtual Communications in the NICU?
  - Not at all confident
  - A little confident
  - Fairly confident
  - Extremely confident
6. Did you receive any education, information, or support for using Virtual Communications in the NICU? (please comment on how we can improve)
  - Yes
  - No
7. Rate your level of agreement with the following statements about your most recent Virtual Communications in the NICU experience:[Table table4-1357633X221081294]
  **Table table4-1357633X221081294:**
  	Strongly agree	Agree	Neutral	Disagree	Strongly disagree	Prefer not to answer
  iPad was easy to set up and use.						
  Zoom was easy to set up and use.						
  Audio quality was good.						
  Video quality was good.						
  Scheduling the session was easy.						
  Length of session was appropriate.						
  I felt confidentiality was maintained.						
  My daily workflow was not impacted.						
8. Please explain your responses to the previous Q7. (particularly if you replied with ‘Strongly agree’ or ‘Strongly disagree’):
9. Rate your level of agreement with the following statements about your experience with Virtual Communications in the NICU:[Table table5-1357633X221081294]
  **Table table5-1357633X221081294:**
  	Strongly agree	Agree	Neutral	Disagree	Strongly disagree	Prefer not to answer
  I perceived a positive impact on the Parent.						
  I perceived a positive impact on the Infant.						
  I believe this experience was beneficial for the entire NICU Care Team.						
10. Please explain your responses to the previous Q9. (particularly if you replied with ‘Strongly agree’ or ‘Strongly disagree’):
11. Rate your overall experience with Virtual Communications in the NICU:
  - Excellent
  - Good
  - Neutral
  - Poor
  - Terrible
12. Would you recommend Virtual Communications in the NICU to families who cannot visit?
  - Yes
  - No
13. Please provide any additional comments, feedback or suggestions for improvement:

## Appendix 2. Parent Survey Questions

1. What gestational age was your infant at birth?
  - 25 weeks or less
  - 26 to 29 weeks
  - 30 to 34 weeks
  - 35 weeks or more
2. How old was your infant when you most recently participated in Virtual Communications in the NICU?
  - First week of life
  - Second week of life
  - During or after the third week of life
3. How many times have you participated in Virtual Communications in the NICU (including the most recent use)?
  - 1
  - 2
  - 3
  - 4 or more
4. What is your age?
  - Less than 18 years
  - 18 to 24 years
  - 25 to 34 years
  - 35 to 44 years
  - 45 years or older
5. What is your relationship to the infant?
  - Mother
  - Father
  - Other caregiver, ie. Grandparent (please specify)
6. In which type of Virtual Communications in the NICU did you most recently participate?
  - Bedside Virtual Visit (w/ infant)
  - Virtual Rounds (w/ medical team)
  - Other Virtual Meeting (specify if possible)
  - I'm not sure
7. For what reason(s) did you use Virtual Communications in the NICU? (select all that apply)
  - Illness (in self or family other than infant)
  - Work
  - Other children to care for
  - Live far from hospital (in Calgary)
  - Live far from hospital (outside Calgary)
  - Transportation issues
  - Financial issues
  - Hospital / unit restrictions
  - Preferred virtual visit instead of in-person visit
  - Other (please specify):

7a. If possible, please explain why you preferred to do virtual visit instead of in-person visit:
8. Did you receive any education or information about using Virtual Communications in the NICU? (please comment on how we can improve)
  • Yes
  • No
9. Rate your level of agreement with the following statements about your most recent Virtual Communications in the NICU experience:[Table table6-1357633X221081294]
  **Table table6-1357633X221081294:**
  	Strongly agree	Agree	Neutral	Disagree	Strongly disagree	Prefer not to answer
  Zoom was easy to set up and use.						
  Audio quality was good.						
  Video quality was good.						
  Scheduling the session was easy.						
  Length of session was appropriate.						
  I felt confidentiality was maintained during the call.						
  This was convenient in my daily life.						
10. Please comment on your responses to the previous
11. Rate your level of agreement with the following statements about your most recent experience with Virtual Communications (VC) in the NICU:[Table table7-1357633X221081294]
  **Table table7-1357633X221081294:**
  	Strongly agree	Agree	Neutral	Disagree	Strongly disagree	Prefer not to answer
  VC helped me better understand my infant's medical issues.						
  VC helped me better understand my infant's care plans.						
  During VC I was able to participate in making decisions about my infant's care plan.						
  VC helped me feel like a valued member of my infant's care team.						
  During VC I felt a bond with my infant.						
12. Please comment on your responses to the previous:
13. Please tell us about any emotions that you experienced directly associated with Virtual Communications in the NICU - not related to your infant's medical condition (select any that apply):[Table table8-1357633X221081294]
  **Table table8-1357633X221081294:**
  	Not at all	Very little	Somewhat	Very much
  Happy				
  Sad				
  Excited				
  Confused				
  Relieved				
  Grateful				
  Anxious				
  Comfortable				
  Calm				
  Angry				
  Overwhelmed				
  Frustrated				
  Other (describe)				
14. Please provide additional comments about your emotional experience with Virtual Communications in the NICU:
15. Did Virtual Communications in the NICU meet your expectations?
   • Yes
   • No
15a. If not, please tell us how we can improve:
16. Would you recommend Virtual Communications in the NICU to other families who cannot visit?
   • Yes
   • No
17. Please rate your overall experience with Virtual Communications in the NICU:
   • Excellent
   • Good
   • Neutral
   • Poor
   • Terrible
18. Please provide any additional comments, feedback or suggestions for improvement:

## Appendix 3. Responses to Likert Scale Questions

Staff Survey Likert Scale Questions and Responses[Table table9-1357633X221081294]

**Table table9-1357633X221081294:**

	Strongly agree	Agree	Neutral	Disagree	Strongly agree	Prefer not to answer	**Total**
iPad was easy to set up and use.	6	5	1	3	1	0	**16**
Zoom was easy to set up and use.	5	5	1	3	2	0	**16**
Audio quality was good.	6	5	2	1	1	1	**16**
Video quality was good.	6	7	2	0	0	1	**16**
Scheduling the session was easy.	6	4	3	2	0	1	**16**
Length of session was appropriate.	7	7	1	0	0	1	**16**
I felt confidentiality was maintained.	7	6	1	1	0	1	**16**
My daily workflow was not impacted.	5	4	4	2	1	0	**16**
I perceived a positive impact on the parent/caregiver.	11	4	1	0	0	0	**16**
I perceived a positive impact on the infant.	6	2	8	0	0	0	**16**
I believe this experience was beneficial for the entire NICU care team.	9	5	2	0	0	0	**16**
**Total**	**74**	**54**	**26**	**12**	**5**	**5**	**176**

Parent Survey Likert Scale Questions and Responses[Table table10-1357633X221081294]

**Table table10-1357633X221081294:**

	Strongly agree	Agree	Neutral	Disagree	Strongly disagree	Prefer not to answer	**Total**
Zoom was easy to set up and use.	6	4	0	0	0	0	**10**
Audio quality was good.	6	3	0	1	0	0	**10**
Video quality was good.	5	3	2	0	0	0	**10**
Scheduling the session was easy.	8	0	1	1	0	0	**10**
Length of session was appropriate.	4	6	0	0	0	0	**10**
I felt confidentiality was maintained.	7	2	1	0	0	0	**10**
This was convenient in my daily life.	7	3	0	0	0	0	**10**
VC helped me better understand my infant's medical issues.	4	3	3	0	0	0	**10**
VC helped me better understand my infant's care plans.	5	2	3	0	0	0	**10**
During VC I was able to participate in making decisions about my infant's care plan.	3	2	3	2	0	0	**10**
VC helped me feel like a valued member of my infant's care team.	5	3	2	0	0	0	**10**
During VC I felt a bond with my infant.	5	3	1	1	0	0	**10**
**Total**	**65**	**34**	**16**	**5**	**0**	**0**	**120**

## Appendix 4. Supplementary Results

Staff survey questions and responses.[Table table11-1357633X221081294][Table table12-1357633X221081294]

**Table table11-1357633X221081294:**

Question	Response				
How confident were you using VC in the NICU?	*Not at all – 0*	*A little – 3*	*Fairly – 7*	*Extremely – 6*	* *
Did you receive any education, information, or support for using Virtual Communications in the NICU?	*Yes – 9*	*No – 7*	* *	* *	* *
Would you recommend Virtual Communications in the NICU to other families who cannot visit?	*Yes – 16*	*No – 0*	* *	* *	* *
Rate your overall experience with virtual communications in the NICU	*Excellent – 6*	*Good – 8*	*Neutral – 1*	*Poor – 0*	*Terrible – 1*

Parent Survey Questions and Responses.

**Table table12-1357633X221081294:**

Question	Response				
Did you receive any education, information, or support for using Virtual Communications in the NICU?	*Yes – 9*	*No – 1*	* *	* *	* *
Did Virtual Communications in the NICU meet your expectations?	*Yes – 9*	*No – 1*	* *	* *	* *
Would you recommend Virtual Communications in the NICU to other families who cannot visit?	*Yes – 10*	*No – 0*	* *	* *	* *
Rate your overall experience with virtual communications in the NICU	*Excellent – 6*	*Good – 3*	*Neutral – 0*	*Poor – 1*	*Terrible – 0*
